# Immune Checkpoint Blockade in Advanced Cutaneous Squamous Cell Carcinoma: What Do We Currently Know in 2020?

**DOI:** 10.3390/ijms21239300

**Published:** 2020-12-06

**Authors:** Anja Wessely, Theresa Steeb, Ulrike Leiter, Claus Garbe, Carola Berking, Markus Vincent Heppt

**Affiliations:** 1 Department of Dermatology, Deutsches Zentrum Immuntherapie (DZI), Universitätsklinikum Erlangen, Friedrich-Alexander-University Erlangen-Nürnberg (FAU), 91054 Erlangen, Germany; anja.wessely@uk-erlangen.de (A.W.); theresa.steeb@uk-erlangen.de (T.S.); carola.berking@uk-erlangen.de (C.B.); 2 Comprehensive Cancer Center Erlangen-European Metropolitan Area of Nuremberg (CCC ER-EMN), 91054 Erlangen, Germany; 3 Department of Dermatology, Eberhard Karls University of Tübingen, 72074 Tübingen, Germany; Ulrike.Leiter@med.uni-tuebingen.de (U.L.); claus.garbe@web.de (C.G.)

**Keywords:** immune checkpoint blockade, cutaneous squamous cell carcinoma, skin cancer, cemiplimab, pembrolizumab

## Abstract

Cutaneous squamous cell carcinoma (cSCC) is the second most common skin cancer that predominantly arises in chronically sun-damaged skin. Immunosuppression, genetic disorders such as xeroderma pigmentosum (XP), exposure to certain drugs and environmental noxae have been identified as major risk factors. Surgical removal of cSCC is the therapy of choice and mostly curative in early stages. However, a minority of patients develop locally advanced tumors or distant metastases that are still challenging to treat. Immune checkpoint blockade (ICB) targeting CTLA-4, PD-L1 and PD-1 has tremendously changed the field of oncological therapy and especially the treatment of skin cancers as tumors with a high mutational burden. In this review, we focus on the differences between cSCC and cutaneous melanoma (CM) and their implications on therapy, summarize the current evidence on ICB for the treatment of advanced cSCC and discuss the chances and pitfalls of this therapy option for this cancer entity. Furthermore, we focus on special subgroups of interest such as organ transplant recipients, patients with hematologic malignancies, XP and field cancerization.

## 1. Introduction

Cutaneous squamous cell carcinoma (cSCC) represents globally the second most common skin cancer after basal cell carcinoma (BCC) [1]. It derives from atypic keratinocytes located in the stratified squamous epithelium in the upper layers of the skin. The accumulation of genetic aberrations is thought to occur in a multistep process leading to the development of actinic keratoses (AK), which eventually progress to cSCC [2], although the risk is presumably low for single lesions. The presence of multiple AK and additional signs of chronic UV damage in the adjacent skin increases the risk for progression considerably [3,4,5]. Reported cSCC incidence rates range from 20 per 100,000 person-years (PYs) in Norway to 387 per 100,000 PYs in Australia [6], and over the last 40 years, the incidence has steadily increased in most countries [7,8]. Although this trend seems to have plateaued in Australia and the US as recently shown by Stang et al., incidence rates still increase strongly with age in all countries [8].

cSCC is most commonly present in elderly people with fair skin and arises particularly at sites that were long-term exposed to UV light, such as the scalp, face, ears and upper extremities [9,10,11]. Immunocompromised patients including solid organ transplant recipients (OTRs) [12], HIV-positive patients [13] and patients with chronic lymphatic leukemia [14,15] also have an increased risk for developing cSCC, indicating that an impaired immune system can promote cSCC development. UV light mediates beneficial effects such as vitamin D production, regulation of the central hypothalamic-pituitary-adrenal axis and increase in β-endorphin levels [16]. However, it contributes to skin carcinogenesis in two different ways. On the one hand, UV-B light causes characteristic transitions of cytosine to thymine in UV-damaged DNA. This mutational pattern is found for instance in *TP53*, the most commonly mutated gene in cSCC, which codes for the tumor suppressor p53 [17]. On the other hand, the immunosuppressive effect of UV-A can also contribute to cSCC formation [18]. Patients with impaired DNA repair caused by rare familial syndromes such as xeroderma pigmentosum (XP) also commonly develop cSCC [19]. Interestingly, a higher number of cSCC has been observed in patients who had received the hedgehog inhibitor vismodegib for BCC therapy or single agent BRAF inhibitors such as vemurafenib for melanoma therapy [20,21]. Moreover, in recent studies, it was observed that AK patients treated with ingenol mebutate developed more cSCC compared to other treatment options and consequently, the European Medicines Agency (EMA) withdrew the marketing authorization of this substance [22,23,24]. Additionally, environmental noxae as arsenic, nitrosamines, alkylating agents and polycyclic aromatic hydrocarbons have been identified as risk factors for developing cSCC [25]. Notably, cSCC arising in previously irradiated or burned skin are usually more aggressive than those arising in sun-damaged skin [26].

Surgical removal of cSCC is the therapy of choice and mostly curative in early stages [27]. However, a subset of patients develops locally advanced tumors or distant metastases that are challenging to treat. Larger high-quality studies and treatment recommendations especially for systemic therapy of advanced cSCC stages are scarce as lymphogenic or haematogenic metastasis only occurs in a minority of patients. Chemotherapy and epidermal growth factor receptor (EGFR) inhibitors have shown limited response as reported in the few available trials [28]. Most of the affected patients are also relatively old and suffer from multiple comorbidities. Hence, patients with locally advanced cSCC and distant metastases still have a very poor prognosis [29,30].

Immune checkpoint blockade (ICB) targeting CTLA-4, PD-L1 and PD-1 have tremendously changed the field of oncological therapy including particularly skin cancers as tumors with a high mutational burden. ICB has not only been successfully applied in melanoma [31] but also in non-small cell lung cancer (NSCLC) [32] and head and neck squamous cell carcinoma (HNSCC) [33]. Thus, the expectations were sufficiently high for a similar efficacy to be achieved in advanced cSCC as well.

In this review, we focus on the differences between cSCC and cutaneous melanoma (CM) and the resulting implications for prognosis, summarize the current evidence on ICB for the treatment of advanced cSCC and discuss the advantages and pitfalls of this therapy option for this cancer entity.

## 2. cSCC vs. CM: Genetic Differences and Implications for Prognosis

The cells of origin of both cSCC and CM reside in the epidermis, the top layer of the skin. Although being located within the same organ, these cells have distinct physiologic functions and are derived from precursor cells of different embryonic origin. cSCC arises from epidermal keratinocytes which are the main cell type building the skin barrier that protects underlying tissues against mechanical stress, chemicals, water loss and infections with viruses, bacteria, fungi and parasites [34]. They terminally differentiate into corneocytes that lack cell organelles and the nucleus and are, therefore, unable to proliferate [34]. Due to their localization, these cells are exposed to a variety of physical and chemical noxae including UV radiation. Melanocytes are pigment-producing cells residing near the basal layer of the epidermis, and they are the cells of origin of melanoma [35]. They are characterized by their typical shape with long protrusions (dendrites) and the presence of melanosomes [36]. These cell organelles contain a brownish pigment composed of one of two types of melanin, eumelanin (brown to black) or pheomelanin (yellow to red), and the ratio of eumelanin:pheomelanin determines the pigmentation of the skin [37,38]. Both melanin pigments are synthesized in a multistep cascade involving several enzymes based on the common precursor dopaquinone that is generated from the amino acid tyrosine [37]. Melanin is distributed by one melanocyte to about 30 surrounding keratinocytes [39], which take up this pigment and drape it around their nuclei as “sunscreen” to protect the DNA from UV-induced damage [35]. Eumelanin mediates the photoprotective effects, whereas degradation of the photolabile pheomelanin leads to the generation of reactive oxygen species including superoxide, hydrogen peroxide and hydroxyl radicals, which can result in additional DNA damage [40,41,42]. Fair-skinned individuals with red hair have a higher proportion of pheomelanin compared to individuals with darker skin and they have a higher risk for developing melanoma and non-melanoma skin cancer (NMSC) [40,43].

UV light stimulates melanin formation (melanogenesis) in a para-, auto- and even intracrine fashion, resulting in tanning of the skin [16]. In particular, exposure to UV-B light increases the expression of several proteins that are involved in pigmentation regulation including the G-protein-coupled melanocortin 1 receptor (MC1R), corticotropin-releasing hormone, urocortin and proopiomelanocortin (POMC) as well as the production of alpha melanocyte-stimulating hormone (α-MSH), adrenocorticotropic hormone (ACTH) and β-endorphin, which are generated by cleavage of their common precursor POMC [16,44]. POMC is mostly produced in the pituitary gland, but also directly in the skin by both keratinocytes and melanocytes and in other peripheral tissues [45,46].

Eumelanin production is stimulated by the binding of α-MSH and ACTH to their receptor MC1R, whereas agouti signaling protein (ASIP) favors the production of pheomelanin by affecting the binding of α-MSH to MC1R [38,40,47]. MC1R activation leads to increased intracellular cAMP levels as well as an increased phosphorylation and activation of CREB transcription factors [38,40]. Together with other transcription factors such as SOX10 and PAX3, they activate the transcription of genes that are involved in melanin synthesis—e.g., *MITF*, the master regulator of pigmentation [38,40]. Transcription factors such as MITF and SOX10 control the expression of enzymes involved in melanogenesis and also play a role in melanoma formation and progression [38]. Apart from POMC and its derivatives, which can also act in melanocytes in an intracrine manner, keratinocytes secrete a variety of factors including prostaglandin E2 and growth factors such as hepatocyte growth factor (HGF), stem cell factor (SCF), fibroblast growth factor (FGF), endothelin-1 in response to UV exposition to stimulate cell proliferation, differentiation and melanogenesis of adjacent melanocytes [41,47]. In addition, melanogenesis can be further influenced via estrogen, adrenergic and muscarinic receptors that are also able to increase intracellular cAMP levels in melanocytes [38,41,48]. Interestingly, keratinocytes and melanocytes are derived from precursor cells of different embryonic origin. Both keratinocytes and melanocytes are cells of ectodermal origin; however, the melanocyte precursors are originally located in the neural crest next to the neural tube. These cells migrate actively into the epidermis of the fetus at 12 weeks of gestation, where they develop into mature melanocytes [49]. The different embryonic origin at least partly explains the distinct aggressiveness and tendency to develop metastases. Cancer cells can re-express proteins that are exclusively expressed during embryogenesis [50]. In the case of melanoma, the tumor cells can acquire a migratory, invading phenotype suitable for forming metastasis by re-expressing proteins which are usually expressed exclusively during skin development. This phenomenon has been demonstrated for instance for the neural crest transcription factors Brn3a and recently for MSX1 [51,52], which both contribute to melanoma progression.

Although keratinocytes and melanocytes are exposed to UV light and environmental noxae in a similar manner, the impact on cSCC and melanoma formation is slightly different. cSCC mostly arises in chronically sun-damaged skin areas after long-term UV exposure over decades, while carcinogenic agents such as polycyclic aromatic hydrocarbons and arsenic can also contribute [6,53]. In contrast, melanoma arises both in sites with intermittent sun exposure [53,54] and in chronically sun-damaged areas. In comparison to cSCC, environmental noxae as well as UV light play a minor role in melanoma pathogenesis. Despite all these differences, both skin cancer entities comprise tumors with a high mutational burden which seems to be a major reason for a good response to ICB [55].

## 3. ICB in cSCC: What Is the Current Evidence?

ICB intends to boost the intrinsic anti-tumor immune response by blocking mechanisms that counteract overwhelming immune responses. The pioneering results in difficult-to-treat cancers such as melanoma and other squamous cell carcinoma including NSCLC and HNSCC have brought ICB into the focus of cSCC research. Over the last couple of years, immune checkpoint-blocking antibodies have been investigated in several trials. The first hints for a probable success of ICB in cSCC were initially described in case reports of melanoma patients coincidentally diagnosed with cSCC, who had received ICB and subsequently responded [56]. Today, most evidence for the efficacy of ICB in advanced cSCC is available for anti-PD-1 antibodies.

### 3.1. Anti-CTLA-4 Antibody: Ipilimumab

Day et al. reported the case of a metastatic cSCC patient who had been diagnosed coincidentally with metastatic BRAF^G469E^-mutated melanoma and, therefore, received ipilimumab every 3 weeks (Q3W, no dosage reported) for 4 cycles [56]. The patient showed a decrease in the cSCC metastases after three cycles of ipilimumab and achieved a partial response (PR) (Table 1). At the time of publication, the progression-free survival (PFS) was 8 months. No adverse events (AEs) ≥ grade 3 were reported. The authors described the quality of life to be excellent.

### 3.2. Anti-PD-1-Blocking Antibodies

#### 3.2.1. Nivolumab

One case series and some case reports represent the only available evidence for the efficacy of nivolumab in cSCC. The retrospective case series described five advanced NMSC patients who were treated with PD-1 blocking antibodies [57]. Four patients had an advanced cSCC and one patient an advanced BCC. Two of the cSCC patients received pembrolizumab 2 mg/kg Q3W and the other two cSCC patients received nivolumab 3 mg/kg every 2 weeks (Q2W). One of the cSCC patients had a stereotactic radiation of a brain lesion while receiving pembrolizumab. PRs were observed in two cSCC patients who had been treated with pembrolizumab and nivolumab, respectively. Stable disease (SD) was observed in all remaining cSCC patients. PFS with pembrolizumab was reported to be ≥4 months and ≥7 months for the two patients and between 6 and at least 7 months for the nivolumab group. No median overall survival (OS), 1-year survival rates or serious AEs were reported. One patient receiving nivolumab died suddenly after 6 months most likely due to arrhythmia; however, the authors did not report whether his death was treatment-related.

Goldman et al. described the case of a kidney transplant recipient who developed multiple cSCC and metastases. Treatment with nivolumab 3 mg/kg led to a PR and a PFS of at least 18 months; however, the patient developed symptoms of an acute kidney injury and allograft rejection, which eventually resulted in the removal of the transplant [58]. The patient described by Oliveira et al. was diagnosed with lymph node and lung metastases from cSCC. Nivolumab also led to a PR, which lasted for at least 12 months [59]. Interestingly, a bone pseudo-progression was observed in several thoracic vertebrae by ^18^F-FDG PET/CT. No AEs ≥ grade 3 were reported.

Fujimura et al. reported the case of a patient with unresectable recurrent cSCC of the scalp with meningeal invasion and cervical lymph node metastases [60]. The patient was pretreated with cetuximab and cisplatin or 5-fluorouracil combination therapy and finally received nivolumab 240 mg Q2W, which led to a complete response (CR) with no signs of recurrence after 1 year at the time of the publication. No AEs were reported.

Oro-Ayude et al. described the case of a metastatic cSCC patient who was pretreated with cisplatin, cervical radiation and methotrexate [61]. He received nivolumab 3 mg/kg Q2W and achieved a CR ten months after the first dose. No AEs ≥ grade 3 were reported.

Two other publications summarized the efficacy of nivolumab and pembrolizumab for advanced cSCC. However, the authors did not distinguish clearly between both treatment regimens when reporting the results. Beasley et al. evaluated the efficacy of nivolumab and pembrolizumab for the treatment of unresectable cSCC in a retrospective study (*n* = 18) [62]. Seventeen patients received nivolumab 3 mg/kg Q2W or every 4 weeks (Q4W) in case a CR or PR were achieved with adverse effects such as fatigue; the remaining patient received pembrolizumab (2 mg/kg) Q3W. Four patients had a CR (22%) and 10 patients a PR (56%), resulting in an objective response rate (ORR) of 78%. Three patients showed SD and one patient progressive disease (PD). The median duration of response (DoR) was 12 months; median PFS and OS as well as 1-year survival rates and AEs ≥ grade 3 were not reported. Another case series from Blum et al. described three cSCC patients who received nivolumab (3 mg/kg) Q2W (*n* = 2) or 200 mg nivolumab Q2W (*n* = 1) [63]. All patients had a PR, and no AEs ≥ grade 3 were observed. Median PFS and OS as well as 1-year survival rates were not reported.

#### 3.2.2. Pembrolizumab

In the prospective phase II study published by Ferrarotto et al., the efficacy of pembrolizumab (200 mg Q3W) in rare unresectable or metastatic cancers including cSCC (NCT02721732) was investigated [64]. Eleven patients with advanced cSCC were enrolled. PR was observed in four patients (36%), SD in one patient (9%) and PD in five patients (45%) within 27 weeks after initiation of the study. One patient had not been evaluated at the time of the publication. No AEs ≥ grade 3 were observed, and no median PFS and OS were reported.

The open-label, single arm, prospective phase II study KEYNOTE-629 (NCT03284424) investigated the efficacy of pembrolizumab (200 mg Q3W) in two cohorts of locally advanced and metastatic or recurrent cSCC. Grob et al. present the data of the first interim analysis of the cohort of recurrent or metastatic cSCC patients [65]. In total, 105 patients received at least one dose of pembrolizumab. CR was observed in 4 patients (3.8%), PR in 32 patients (30.5%), SD in 31 patients (29.5%) with 19 patients lasting for at least 12 weeks. Two patients were not evaluable and 8 patients were not assessed. Six patients (5.7%) developed treatment-related AEs of grade 3–5 and one patient died due to cranial nerve neuropathy which was considered treatment-related. In total, 28 patients (26.7%) interrupted and 13 patients (12.4%) discontinued the therapy due to AEs.

An open-label, prospective, single group, phase II study (NCT02964559) investigated pembrolizumab (200 mg Q3W) for up to 2 years in 11 patients with advanced cSCC [66]. CR was observed in two patients (18%) and PR in four patients (36%), resulting in an ORR of 54%. One patient (9%) had an SD and four patients (36%) a PD. The authors reported a 6-month PFS of 72% for evaluable patients and 50% of the patients that responded had a durable response of 18 months or greater at the time of data cut-off. Three AEs ≥ grade 3 were documented (hepatitis and pneumonitis).

The CARSKIN trial (NCT02883556), an open-label, single group, prospective phase II study, evaluated pembrolizumab (200 mg Q3W) in treatment-naive patients with advanced cSCC for up to 2 years [67]. Initially, 39 patients were enrolled in the study (primary cohort) and due to the promising interim results, 18 additional patients were recruited (expansion cohort). After 15 weeks, 3 CR (8%) and 13 PR (33%) were achieved in the primary cohort and 1 CR (8%) and 7 PR (39%) were observed in the expansion cohort. Survival data were only provided for the primary cohort. Median PFS was 6.7 months, median OS was 25.3 months and 1-year OS was 75.5%. Treatment-related AEs occurred in 71% of patients. Four patients (7%) had treatment-related AEs of grade ≥3 (colitis, diarrhea, cutaneous vasculitis and cholestasis).

Hermel et al. reported the results of an expanded access program (EAP) investigating pembrolizumab 2 mg/kg Q3W in 8 patients [68]. Half of the patients had a PR and no AEs ≥ grade 3 were observed. Other outcomes were not reported.

Lavaud et al. retrospectively evaluated the response of 4 patients with locally advanced cSCC [69]. They had received pembrolizumab 2 mg/kg Q3W until disease progression, unacceptable toxicity or the physician’s choice to discontinue treatment. Two patients achieved a CR and two patients PD. The median PFS was 14.4 months and the median OS was 15.6 months. No AEs were reported.

Several publications present the cases of patients with advanced cSCC who were successfully treated with pembrolizumab. Lipson et al. presented the case of a kidney-transplanted metastatic cSCC patient who was treated with pembrolizumab (2 mg/kg Q3W) [70,71]. The patient completely responded but developed an acute allograft rejection after 2 months of therapy with pembrolizumab and the kidney had subsequently to be removed. More than 4 years after the removal of the allograft, the patient was still tumor-free and underwent a second kidney transplantation and modified immunosuppression. At the time of the publication, the patient was relapse-free and the allograft had not been rejected for more than 10 months. In another patient with unresectable cSCC, a CR was observed with pembrolizumab 2 mg/kg Q3W [72]. The patient did not develop any AEs ≥ grade 3. Interestingly, the tumor harbored a mutation in the *MLH1* gene, leading to an impaired DNA mismatch repair. Chang et al. described the case of a patient with unresectable cSCC [73]. The patient received pembrolizumab (2 mg/kg) Q3W, which led to a CR. No AEs ≥ grade 3 were reported in this publication; however, in the follow-up report of this case, the authors reported the occurrence of AEs ≥ grade 3, namely fatigue caused by endocrine hypofunction [74]. The duration of response of this patient was 21 months at the time of publication and still ongoing as described by Tran et al., who—in addition to this case—reported on the results of another five patients with advanced cSCC receiving pembrolizumab (2 mg/kg, Q3W, *n* = 4) or nivolumab (3 mg/m^2^, Q2W) [74]. One of the five patients had a CR (20%), three patients a PR and one patient receiving pembrolizumab a PD. PFS was 12 months for the patient treated with nivolumab and ranged from 3 to 10.5 months for pembrolizumab—median OS was not reported. One patient suffered from severe AEs; however, the authors did not mention the grade of severity. Deinlein et al. [75], Stevenson et al. [76], Degache et al. [77], Steineck et al. [78] and Ma et al. [79] reported the cases of six patients with advanced cSCC who received pembrolizumab (2 mg/kg, Q3W). The XP patient described by Deinlein et al. had a PR, which was still ongoing at the time of publication [75]. Similarly, a durable CR was also observed in the patient reported by Stevenson et al., which was still ongoing after 11 months of maintenance therapy. Both patients mentioned in Degache et al. had a PR [77]. Another XP patient successfully treated with pembrolizumab was presented by Steineck et al. [78]. The 7-year-old girl with metastatic cSCC received pembrolizumab and achieved a long-term PR. Ma et al. described the case of a metastatic cSCC patient successfully treated with pembrolizumab [79]. The patient completely responded and was disease-free for 17 months at the time of publication. No publications reported any AEs ≥ grade 3, and all patients were still alive at the time of publication.

Pembrolizumab also seems to be effective for the treatment of locally advanced cSCC as demonstrated by the following three case reports. Cristancho et al. reported the case of a patient with unresectable, locally advanced cSCC who received pembrolizumab 200 mg Q3W and achieved a CR for at least 1 year [80]. No AEs were observed. Delaitre et al. described the case of a patient with locally advanced cSCC on the scalp and BCC on the nose who received pembrolizumab 2 mg/kg Q3W [81]. A CR of the cSCC and BCC was observed after 11 cycles and 7 cycles, respectively. No AEs ≥ grade 3 were reported. Khaddour et al. reported the case of a patient with cutaneous T-cell lymphoma (CTCL) who received allogeneic hematopoietic stem cell transplantation (allo-HCT) and subsequently developed locally advanced cSCC and lymph node metastases [82]. Pembrolizumab 200 mg Q3W was administered, and the patient achieved a CR of cSCC and CTCL for at least 24 months. Pruritus and erythema caused by the CTCL significantly improved and graft-versus-host disease was not worsened. Only grade 1 AEs were reported (macular rash).

#### 3.2.3. Cemiplimab

Migden et al. presented the results of a prospective open-label, multicenter phase I (NCT02383212) and a phase II trial (NCT02760498) evaluating cemiplimab for the treatment of advanced cSCC in 85 patients [83]. Twenty-six patients with locally advanced or metastatic cSCC were enrolled in the expansion cohorts of the phase I study and 59 patients with regional or distant cSCC metastases in the metastatic-disease cohort of the phase II study. The primary outcomes of the phase I study were the safety and AE profile, while the response rate assessed by independent central review was the primary outcome of the phase II study. The secondary outcomes in both studies included PFS, OS, DOR and toxic effects of cemiplimab. In both studies, cemiplimab (3mg/kg Q2W) was applied for up to 48 weeks (phase I) or 96 weeks (phase II) unless stopped due to disease progression or non-tolerable toxic effects.

In the phase I study, 13 patients (50%) had a PR, six patients showed a SD (23%) and three patients progressed (12%), resulting in an ORR of 50% (95% confidence interval (CI) 30–70). No CR was observed and three patients were not evaluable. Seven patients had a duration of response of more than 6 months. Six patients (23.1%) developed AEs of ≥ grade 3, but only two of them (7.7%) were considered to be treatment-related. During the phase I study, one patient died due to renal failure that was considered unrelated to the study treatment, and 3 patients died due to PD. Another patient who discontinued the treatment was lost to follow-up and died due to an unknown cause.

In the phase II study, the ORR was 47% (95% CI 34- 61). Four CR (7%) and 24 PR (41%) were achieved; nine patients (15%) had a SD and eleven patients (19%) a PD. Seven patients could not be evaluated. The median PFS and OS were not reached at the time of the data cut-off. The estimated probabilities of 1-year PFS and 1-year OS were 53% (95% CI 37–66) and 81% (95% CI 68–89), respectively. AEs of ≥ grade 3 were observed in 17 patients (29%) and were considered as treatment-related in five patients (8.5%). Altogether, eleven patients died during the study—eight patients due to the progression of the disease and three patients due to AEs. However, they were not classified as cemiplimab-related by the authors.

In a second cohort of the phase II study (NCT02760498), 78 patients with locally advanced cSCC were enrolled and received cemiplimab (3 mg/kg Q2W) [84]. The ORR by central review was 43.6% (95% CI 32.4–55.3) with 10 CR (13%) and 24 PR (31%). The median PFS and OS were not reached. Treatment-related AEs of ≥ grade 3 were observed in 10.3% of patients (8/78) and the death of one patient was considered as treatment-related. Recently, Rischin et al. published outcomes of another subgroup of this phase II study (NCT02760498) including 56 patients with metastatic cSCC who received fixed dose cemiplimab 350 mg Q3W [85]. The ORR was 41.1% (95% CI 28.1–55.0), and 3 CR (5%), 20 PR (36%), 8 SD (14%) and 14 PD (25%) were observed. The median PFS and OS had not been reached at the time of the publication. The estimated median PFS was 10.4 months, and the estimated 1-year OS was 76.1% (95% CI 56.9–87.6%). In total, 22 patients (39%) developed AEs ≥ grade 3, and 7 of them (12.5%) were considered treatment-related.

Escobar et al. described the case of a patient with both advanced cSCC and metastatic NSCLC who received cemiplimab 3 mg/kg Q2W and radiotherapy of the scalp and cervical region to control local bleeding [86]. The patient responded partially and the only remaining lesion was later excised. Secondary adrenal insufficiency was mentioned as AE, but the severity was not clearly reported. At the time of publication, there had been no tumor progression evident for 8 months.

### 3.3. Combined CTLA-4 and PD-1 Blockade

CTLA-4 and PD-1 blockade have been successfully combined in the past—e.g., for the treatment of advanced melanoma, achieving better results than the corresponding monotherapies [87,88,89]. However, dual ICB also increases the frequency of severe AEs [90].

Evidence for combined ICB in cSCC is sparse. Miller et al. reported the case of a kidney transplant recipient who developed metastatic cSCC 3 years after transplantation [91]. The patient had received a combination of ipilimumab and nivolumab after unsuccessful pretreatment with multiple other therapies. The precise dosage scheme was not indicated. The treatment led to a PR; however, the patient developed signs of an acute kidney injury, which led to the removal of the transplant less than 2 weeks after ICB initiation. The patient died 5 months after starting the immunotherapy during dialysis due to a sudden cardiac arrest that was not considered to be treatment-related and instead more likely to be a secondary complication of his long-term diabetes.

The case of another kidney transplant recipient was described by Trager et al. [92]. The patient developed metastatic cSCC after previous diagnoses of melanoma and multiple NMSC. After unsuccessful pretreatment with cetuximab and cisplatin, the patient was included in a single-arm, open-label, phase I study (NCT03816332) and received cemiplimab (350 mg Q3W) but showed disease progression and therapy was switched to combined ICB. Ipilimumab (3 mg/kg) and nivolumab (1 mg/kg) were administered and the patient partially responded. He developed a rash but otherwise tolerated the therapy well. No signs of allograft rejection were observed.

## 4. ICB in cSCC: The New Standard of Care?

The currently available evidence is still limited, but the results indicate that ICB also seems to be effective in cSCC. ICB generally achieves the highest response rates in tumors with high mutational burdens [55,93]. These tumors are more likely to express neoantigens that can be recognized by cytotoxic T lymphocytes (CTLs) as “foreign”, eventually leading to the lymphocyte-mediated killing of the tumor cells [94]. cSCC has one of the highest mutational burdens among all cancer entities and, therefore, might be a promising candidate for ICB [55,95,96]. However, predicting the success of ICB in cancer is challenging and even in cancer entities harboring an extremely high number of mutations, ICB fails in a subset of patients.

Furthermore, PD-L1 expression levels have been investigated in the past as potential biomarkers for predicting the outcome of ICB [97]. PD-L1-expressing tumors were associated with a better response in several trials investigating anti-PD-1 and anti-PD-L1 antibodies [98]. However, PD-L1 is not an ideal biomarker for predicting response to ICB. Determining tumor positivity by immunohistochemical staining is challenging as both tumor cells and infiltrating immune cells can express PD-L1, which seems to have a different impact on the prognosis [97,98]. Furthermore, expression can vary over time and is often heterogeneously distributed in the tumor tissue; thus, small biopsies may miss PD-L1 expressing cells [97,99]. These uncertainties have led to distinct cut-off values in clinical trials for defining PD-L1 positive tumors which are eligible for ICB, ranging from 1% up to 50% PD-L1 expressing tumor cells [100,101]. PD-L1 expression in cSCC has been evaluated in several retrospective analyses. Varki et al. observed that 26% of the investigated cSCC patients in their study had PD-L1-expressing tumors [102], and 20% of high-risk cSCC and 41% of metastatic cSCC evaluated by Slater and Googe had PD-L1-positive tumor cells [103]. Similarly, García-Díez et al. observed that 26% of non-metastatic cSCC and 50% of metastatic cSCC had at least 1% PD-L1- expressing cells, indicating that advanced cSCC are more likely to express PD-L1 [104]. These data are in line with a recent case series published by Wu et al. who detected PD-L1 expression in 85% of locally advanced and 100% of metastatic cSCC [105].

In addition to PD-L1 expression, the number of tumor-infiltrating immune cells is an important factor that influences response to ICB. Modulating T cells, which have already entered the tumor stroma, will more likely provoke an effective anti-tumor immune response. Immunologically “hot” tumors with a high number of CD3^+^ and CD8^+^ T cells at the tumor center are more susceptible to agents targeting CTLA-4, PD-L1 and PD-1 that modulate T cell function [106,107]. T cell infiltration in cSCC has not been extensively studied yet. However, Wu et al. detected tumor-infiltrating lymphocytes in all investigated samples, suggesting that cSCC may be a “hot” tumor suitable for successful ICB [105].

Based on the results of several phase II trials, the U.S. Food and Drug Administration (FDA) has recently approved cemiplimab and pembrolizumab for the treatment of locally advanced and metastatic cSCC [108,109]. In Europe, only cemiplimab is currently approved [110]. Cemiplimab and pembrolizumab appear to be the most evidence-based substances in this setting, as they were investigated in studies of better quality compared to the remaining publications. They achieved ORR ranging from 36 to 54% for pembrolizumab [65,66] and 41 to 50% for cemiplimab [83,84,85]. At the time of publication, the median PFS reported for pembrolizumab ranged from 6.7 to 6.9 months, and the median OS was 25.3 months in one study and was not reached in another trial [65,67]. Both median PFS and OS were not reached in the studies investigating cemiplimab [83,84,85]. Reported 1-year OS rates ranged from 60 to 76% with pembrolizumab [65,67] and from 76 to 93% with cemiplimab [83,84,85]. In addition to cemiplimab and pembrolizumab, other immune checkpoint-blocking antibodies also showed promising clinical results with objective and durable responses of at least 18 months [58]. However, most of the publications were selected case reports, being at high risk of publication bias. Furthermore, the quality of evidence is limited as ICB in cSCC patients has not been investigated in randomized controlled trials (RCTs) yet. Hence, the evidence for the use of ICB in cSCC is still limited compared to other entities such as melanoma. However, 27 trials investigating ICB in cSCC are currently ongoing (Table 2).

This lack of RCTs must be considered in future treatment guidelines which currently fall short of providing specific recommendation for ICB in advanced cSCC [111]. Nevertheless, due to the durable nature of the responses and the lack of treatment alternatives, ICB should receive a high grade of recommendation and should be discussed in the first treatment line in unresectable or metastatic disease.

## 5. ICB for Special cSCC Subgroups: Organ Transplant Recipients, Patients with Hematologic Malignancies, Xeroderma Pigmentosum, AK with Field Cancerization

In recent decades, the number of solid OTRs has been steadily rising. In 2018, 3595 transplantations were conducted in Germany, and there were 146,751 transplantations globally [112]. OTRs require immunosuppressing medication in order to prevent allograft rejection. However, constant immunosuppression not only dampens the immune response against the transplant but also impairs the response against precancerous cells. OTRs are at a 2- to 6-fold increased risk for developing tumors compared to the overall population [113] and a more than 100-fold increased risk for developing cSCC [114]. Unlike immunocompetent cSCC patients, OTRs more often develop multiple primary tumors as well as locally advanced and metastatic disease [115]. Treating advanced cSCC is challenging and the immune checkpoint-blocking agent cemiplimab can cause allograft rejection as it causes an activation of the immune system. However, patients may be willing to choose the lesser of two evils in certain circumstances. In the case of kidney transplant recipients, the loss of the transplanted organ will significantly diminish their quality of life, but it may be successfully compensated by hemodialysis. However, allograft rejection will certainly be life-threatening for patients who have received other organs such as heart and lung whose function cannot be permanently compensated by medical devices currently. On the other hand, effective alternative options for treating advanced cSCC are not available, highlighting the difficulties of choosing an appropriate therapy. Nevertheless, cemiplimab has been successfully used in OTRs in the past, as demonstrated by several case reports (Table 1). The results indicate that ICB is effective in these patients and the anti-tumor response does not seem to be compromised by previous immunosuppression or prednisone medication during ICB [58,71,92]. However, high-quality evidence is also lacking for this group of patients, and evidence on the efficacy is restricted to anecdotal reports. RCTs would also be desirable in this setting but most likely will not be conducted due to the limited number of affected patients. Thus, its use in OTRs remains controversial.

Nucleotide excision repair (NER) is a mechanism essentially required for the repair of bulky DNA alterations caused by UV light and other mutagens [116]. Mutations affecting the seven XP genes *XPA* to *XPG* or *POLH* which are part of the NER mechanism lead to XP, a rare genetic disorder that leads to the accumulation of mutations over time due to a permanently impaired NER. As a result, XP patients are at high risk for developing multiple melanoma and NMSC lesions including cSCC in sun-exposed body sites already at an early age [117]. No therapy for restoring NER function is available at the moment; thus, XP therapy focuses on the treatment of existing skin cancer lesions and their prevention. Surgical excision of multiple skin tumors and advanced disease may not always be the most suitable option. The case reports described above have demonstrated that ICB may be a suitable systemic therapy option for XP patients affected by advanced, unresectable cSCC [75,78]. In addition, other case reports which did not clearly distinguish between cSCC and other skin tumors and, therefore, were not included in the list above, underline the efficacy of ICB against melanoma and NMSC [118,119,120,121].

While ICB has been used for the treatment of cSCC, little is known of the efficacy against precancerous lesions such as AK. AK are keratinocytic lesions arising in chronically sun-damaged areas that eventually progress to invasive cSCC [3,122]. Orloff et al. presented the impact on NMSC and AK in a case series of three patients who received ICB for other indications [123]. One of the cases described in this series was a patient with metastatic melanoma and extended AK. After receiving two doses of ipilimumab followed by two doses of pembrolizumab, all AK lesions disappeared. The authors did not comment on the efficacy of ICB against the metastatic disease in this patient. Although this report suggests that ICB can clear AK lesions, other physicians (personal communication) and we could not observe such effects in our patients. Anyhow, ICB will hardly ever be used as a treatment against AK as several other efficient and more favorable options in terms of safety profile exist. Thus, AK may be cleared by ICB only as a side effect, if at all.

Recently, Leiter et al. reported the results of a retrospective analysis evaluating the efficacy of ICB in non-resectable melanoma, cSCC and Merkel cell carcinoma with and without concomitant hematological malignancies [124]. Seventy-five of the included patients had unresectable, advanced cSCC and received either nivolumab or pembrolizumab. Fifteen cSCC patients had a concomitant hematological malignancy, most of them chronic lymphatic leukemia (*n* = 8) and other types of non-Hodgkin lymphoma (*n* = 5). In this subgroup, one patient achieved a CR and three patients a PR, resulting in an ORR of 26.7%. The median PFS and OS were 4.0 months (95% CI 0.3 to 7.9) and 14.9 months (95% CI 0.1 to 31.2), respectively. In the subgroup of cSCC patients without hematological malignancies, the ORR was 33.8% with eight patients achieving a CR and twelve a PR. Here, the median PFS and OS were not reached at the time of publication. There were no statistically significant differences between both cSCC subgroups except for the PFS (*p* = 0.002). AEs were not reported in this publication.

## 6. Conclusions

Advanced cSCC is a major therapeutic challenge and still difficult to treat. In the past, several treatment variations have failed to demonstrate their anti-tumor efficacy. This has now at least partly changed. Although the evidence is still limited, ICB has achieved very promising results in this cancer entity, finally leading to the recent approval of the anti-PD-1 antibodies cemiplimab in Europe and the U.S. and pembrolizumab in the U.S. With these antibodies, we have important tools now to treat advanced cSCC also in difficult-to-treat patients. Promising results have also been observed in special patient groups such as OTRs and XP patients. Nevertheless, future high-quality RCTs will be required to underline the efficacy of ICB in this cancer entity.

## Figures and Tables

**Table 1:** Immune checkpoint blockade in cutaneous squamous cell carcinoma (cSCC) patients.

Author, Year [Reference]	Design	*n*	Intervention	ORR	PR	CR	PFS (Months)	OS (Months)	1-Year OS	AEs ≥Grade 3
Day 2017 [56]	Case report	1	Ipi *	1/1	1/1	0/1	≥8	-	-	0
Borradori 2016 [57]	Case series	2	Pem 2 mg/kg	1/2	1/2	0/2	≥4, ≥7	-	-	0
		3	Nivo 3 mg/kg	1/2	1/2	0/2	6 to ≥7	-	-	0
Beasley 2017 [62]	Case series	17	Nivo 3 mg/kg	14/18	10/18	4/18	-	-	-	3/18 (fatigue, haemolytic anemia, colitis)
		1	Pem 2 mg/kg							
Blum 2018 [63]	Case series	2	Nivo 3 mg/kg	3/3	3/3	0/3	-	-	-	0
		1	Nivo 200 mg fix							
Goldman 2018 [58]	Case report	1 (OTR)	Nivo 3 mg/kg	1/1	1/1	0/1	≥18	-	-	allograft rejection
Fujimura 2020 [60]	Case report	1	Nivo 240 mg fix	1/1	0/1	1/1	-	-	-	-
Oliveira 2018 [59]	Case report	1	Nivo 3 mg/kg	1/1	1/1	0/1	≥12	-	-	0
Oro-Ayude 2020 [61]	Case report	1	Nivo 3 mg/kg	1/1	0/1	1/1	-	-	-	0
Assam 2016 [72]	Case report	1	Pem 2 mg/kg	1/1	0/1	1/1	-	-	-	0
Chang 2016 [73]	Case report	1	Pem 2 mg/kg	1/1	0/1	1/1	-	-	-	unclear
Cristancho 2020 [80]	Case report	1	Pem 200 mg fix	1/1	0/1	1/1	-	-	-	0
Degache 2018 [77]	Case series	2	Pem 2 mg/kg	2/2	2/2	0/2	-	-	-	0
Deinlein 2017 [75]	Case report	1 (XP patient)	Pem 2 mg/kg	1/1	1/1	0/1	-	-	-	0
Delaitre 2020 [81]	Case report	1	Pem 2 mg/kg	1/1	0/1	1/1	-	-	-	0
Ferrarotto 2017 [64] NCT02721732	Phase II	11	Pem 200 mg fix	4/11	4/11	0/11	-	-	-	0
Grob 2020 [65] KEYNOTE-629 NCT03284424	Phase II	105	Pem 200 mg fix	36/105	32/105	4/105	6.9	n.r.	60.3%	6 (5.7%, grade 3–5)
Hermel 2018 [68]	EAP	8	Pem 2 mg/kg	4/8	4/8	0/8	-	-	-	0
Khaddour 2019 [82]	Case report	1 (allo-HCT)	Pem 200 mg fix	1/1	0/1	1/1	≥ 24	-	-	0
Lavaud 2019 [69]	Retrospective analysis/case series	4	Pem 2 mg/kg	2/4	0/4	2/4	14.4	15.6	-	0
Lipson 2016/2020 [70,71]	Case report	1 (OTR)	Pem 2 mg/kg	1/1	0/1	1/1	-	-	-	allograft rejection
Maubec 2020 [67] CARSKIN trial NCT02883556	Phase II	39 (primary cohort)	Pem 200 mg fix	16/39	13/39	3/39	6.7	25.3	75.5%	4 (7%)
		18 (expansion cohort)	Pem 200 mg fix	8/18	7/18	1/18	-	-	-	-
Ma 2020 [79]	Case report	1	Pem 2 mg/kg	1/1	0/1	1/1	≥17	-	-	0
Steineck 2019 [78]	Case report	1 (XP patient)	Pem	1/1	1/1	0/1	≥18	-	-	-
Stevenson 2017 [76]	Case report	1	Pem 2 mg/kg	1/1	0/1	1/1	-	-	-	0
Tran 2017 [74]	Case series	4	Pem 2 mg/kg	3/4	2/4	1/4	3 to 10.5	-	-	0
		1	Nivo 3 mg/m^2^	1/1	1/1	0/1	12	-	-	0
Yushak 2019 [66] NCT02964559	Phase II	11	Pem 200 mg fix	6/11	4/11	2/11	≥6	-	-	3 AEs reported
Escobar 2020 [86]	Case report	1	Cem 3 mg/kg Q2W	1/1	1/1	0/1	≥8	-	-	unclear
Migden 2018/2020, Rischin 2020 [83,84,85] NCT02383212 (phase I) NCT02760498 (phase II)	Phase I (la)	26	Cem 3 mg/kg	13/26	13/26	0/26	-	-	-	6/26
	Phase II (m)	59		28/59	24/59	4/59	n.r.	n.r.	81%	17/59
	Phase II (la)	78		34/78	24/78	10/78	n.r.	n.r.	93%	8/78
	Phase II (m)	56	Cem 350 mg fix	23/56	20/56	3/56	n.r.	n.r.	76.1%	7/56
Miller 2017 [91]	Case report	1 (OTR)	Ipi and nivo *	1/1	1/1	0/1	-	-	-	allograft rejection
Trager 2020 [92]	Case report	1 (OTR)	Ipi 3 mg/kg and nivo 1 mg/kg	1/1	1/1	0/1	≥9	-	-	unclear

**Note:** - = not reported; EAP = expanded access program; ORR = overall response rate; PR = partial response; CR = complete response; PFS = progression-free survival; OS = overall survival; n.r. = not reached; AEs = adverse events; OTR = organ transplant recipient; XP = xeroderma pigmentosum patient; allo-HCT = allogeneic hematopoietic cell transplantation; la = locally advanced; m = metastatic; ipi = ipilimumab; pem = pembrolizumab; nivo = nivolumab; cem = cemiplimab; AE = adverse events; * no dosage reported in the studies.

**Table 2:** Ongoing trials investigating immune checkpoint blockade (ICB) in advanced cSCC.

Study ID	Study Design	Start	End *	Sample Size [*n*]	Intervention	Primary Outcomes	Secondary Outcomes	Funding
NCT03901573	Multicenter, open-label, phase Ib	Dec 2019	May 2024	24	Atezolizumab i.v.+ efineptakin alfa (hIL-7-hyFc) i.m. Dose escalation	Safety and tolerability	ORR, DCR, DOR, PFS, OS	NeoImmuneTech Immune Oncology Network
	Multicenter, open-label, two armed, phase IIa			60 (A = 24, B = 36)	Atezolizumab i.v.+ efineptakin alfa (rhIL-7-hyFc) i.m. A: ICB-refractory cSCC B: ICB-naïve cSCC Dose expansion			
NCT03108131	Single-center, single-arm, open-label, phase II	Apr 2017	Jul 2020	60	Atezolizumab i.v. Q2W + cobimetinib p.o. QD on days 1–12, 1 cycle = 28 days, until disease progression or unacceptable toxicity	ORR	PFS	M.D. Anderson Cancer Center National Cancer Institute (NCI)
NCT03944941	Multicenter, open-label, randomized, two-armed, phase II	May 2019	Dec 2023	59	A: Avelumab i.v. on days 1 and 15, up to 24 cycles (1 cycle = 28 days), until disease progression or unacceptable toxicity; patients with avelumab failure will crossover to arm B B: Avelumab + cetuximab: and avelumab i.v. on days 1 and 15 + cetuximab i.v. on days 1, 8, 15, and 22, up to 24 cycles until disease progression or unacceptable toxicity	PFS	ORR, clinical benefit rate, OS, AEs	Alliance for Clinical Trials in Oncology National Cancer Institute (NCI)
AliCe Trial EudraCT: 2018-001708-12	Multicenter, open-label, single-arm, phase II	un-clear	un-clear	52	Avelumab i.v. + cetuximab i.v., intervals and dosage not reported	ORR	PFS, DOR, OS, AEs, QoL	Alcedis GmbH
UNSCARRed trial NCT03737721	Single-center, single-arm, open-label, phase II	Apr 2019	Jun 2023	20	Avelumab i.v. Q2W, first dose 14 days prior to radiotherapy, then 63–66 Gy radiation over 30 daily fractions + avelumab i.v. Q2W for 4 cycles	ORR	PFS, clinical and pathological response rate, AEs	AHS Cancer Control Alberta EMD Serono Alberta Cancer Foundation
NCT03889912	Multicenter, single-arm, open-label, phase I	Apr 2019	Feb 2022	36	Cemiplimab i.t. QW for 12 weeks neoadjuvant, then surgical excision	AEs	ORR, CR rate, pathological response rate, drug concentration over time, anti-drug antibodies	Regeneron Pharmaceuticals Sanofi
NCT04154943	Multicenter, single-arm, open-label, phase II	Mar 2020	Dec 2024	76	Cemiplimab i.v. Q3W	Pathologic CR rate	Major pathologic response, ORR, event-free survival, DFS, OS, AEs, incidence of deaths	Regeneron Pharmaceuticals Sanofi
NCT03969004 (EudraCT: 2019-000566-38)	Randomized, multicenter, two-armed, double-blind, phase III	Jun 2019	Feb 2026	412	Surgery and radiation therapy followed by A: Cemiplimab i.v. B: Placebo i.v. Intervals and dosage not reported	DFS	OS, freedom from locoregional and distant recurrence, cumulative occurance of second primary cSCC, AEs, incidence of deaths	Regeneron Pharmaceuticals Sanofi
NCT04242173	Single-center, single-arm, open-label, phase II	Jan 2020	Jan 2023	27	Cemiplimab i.v., intervals and dosage not reported	ORR	PFS, OS	Regeneron Pharmaceuticals Sanofi
CERPASS trial NCT04050436	Randomized, multicenter, two-armed, open-label, phase II	Oct 2019	Mar 2024	240	A: Cemiplimab i.v. Q3W B: Cemiplimab i.v. Q3W+ RP1 i.t. Q3W	ORR	DOR, PFS, CR rate, OS, AEs, response injected vs. non-injected lesions	Replimune Inc. Regeneron Pharmaceuticals
CONTRAC trial NCT04339062	Non-randomized, single-center, open-label, phase I	Jul 2020	Jul 2022	12 A: allo-HCT B: kidney trans-plant reci-pients	A: Cemiplimab i.v. Q3W B: Cemiplimab i.v. Q3W + everolimus/sirolimus 7–10 days prior to cemiplimab start, then QD + prednisone 40 mg p.o. 1 day prior to cemiplimab start, then QD at tapering doses	Dose-limiting toxicity (A: GVHD, B: allograft rejection)	PFS, OS, ORR, therapeutic response rate, secondary infection rate,	Dana-Farber Cancer Institute Regeneron Pharmaceuticals
NCT02760498 (EudraCT: 2016-000105-36)	Multicenter, open-label, phase II	Apr 2016	Dec 2025	433	Cemiplimab i.v. A: mSCC, Q2W B: laSCC, Q2W C: mSCC, Q3W D: mSCC or laSCC, Q4W E: mSCC or laSCC, Q3W	ORR	DoR, PFS, OS, CR rate, QoL, AEs, pharmacokinetics, correlation PD-L1 expression and ORR/DoR/PFS	Regeneron Pharmaceuticals
NCT04428671	Single-center, open-label, phase I	May 2020	Oct 2030	20	Cemiplimab i.v. Q3W neoadjuvant for up to 3 cycles prior to surgery, then cemiplimab i.v. Q3W adjuvant, starting within 2–6 weeks after surgery or radiation therapy, up to 18 cycles	Pathologic RR	Time to local and systemic recurrence, OS, RFS	Emory University
NCT04315701	Multicenter, open-label, phase II	Jun 2020	Jan 2023	34	Cemiplimab i.v. Q3W up to 3 cycles neoadjuvant, then surgery within 6 weeks after last dose	Pathologic PR	Pathologic CR rate, ORR, PFS, AEs	M.D. Anderson Cancer Center National Cancer Institute (NCI)
NCT03684785	Multicenter, open-label, phase Ib	Dec 2018	Jun 2023	130	Cavrotolimod i.t. twice (dosage and intervals not reported), then with pembrolizumab 2 mg/kg i.v. Q3W Dose escalation	AEs	Finding recommended dose of cavrotolimod for phase II trial ORR, biomarkers	Exicure, Inc.
	Phase II				Cemiplimab 350 mg i.v. Q3W + cavrotolimod i.t. Dose expansion			
NCT04305795	Open-label, phase I/II	Sep 2020	Jun 2024	54	Cemiplimab 350 mg i.v., up to 24 months + ASP-1929 (EGFR antibody-dye conjugate for photoimmunotherapy light treatment)	AEs, ORR	OS, PFS, DOR	Rakuten Medical, Inc.
NCT03816332	Multicenter, single-arm, open-label, phase I	Feb 2019	May 2021	16 kidney trans-plant reci-pients	Tacrolimus p.o. BID + prednisone p.o. QD, within 28 days: Nivolumab i.v. Q4W, up to 24 cycles until disease progression or unacceptable toxicity. Patients with PD at 16 weeks: Nivolumab i.v. + ipilimumab i.v. Q3W + tacrolimus p.o. BID + prednisone p.o. QD, up to 4 cycles until disease progression or unacceptable toxicity. Starting 6 weeks later, patients receive nivolumab i.v. Q4W, up to 21 cycles until disease progression or unacceptable toxicity.	CR, PR and SD rate, patients without allograft loss	ORR, allograft rejection rate, DOR (CR and PR), PFS, OS	National Cancer Institute (NCI)
CA209-9JC trial NCT03834233	Single-center, open-label, phase II	Sep 2019	Dec 2022	24	Nivolumab 3 mg/kg i.v. Q2W until disease progression or up to 12 months	ORR	AEs, PFS	Instituto do Cancer do Estado de São Paulo
NCT04204837	Multicenter, open-label, phase II	Mar 2017	Dec 2023	31	Nivolumab 240 mg i.v. Q2W until disease progression, unacceptable toxicity or up to 2 years	ORR	DCR, DOR, PFS, OS	Salzburger Landeskliniken Bristol-Myers Squibb
NCT02978625	Multicenter, open-label, phase II	Sep 2017	Jun 2021	68	T-VEC i.t. on day 1, if no response: Nivolumab i.v. Q3W (first cycle), then Q2W until disease progression, unacceptable toxicity or up to 1 year	RR to T-VEC alone, ORR to T-VEC + nivolumab	Durable RR, RR of injected and non-injected lesions, PFS, OS, AEs	National Cancer Institute (NCI)
Pelican trial NCT03773744	Open-label, phase I	Jan 2020	Dec 2021	40	A: Cyclophosphamide 300mg/m^2^ 3 days prior to Ad-MAGEA3 fixed dose i.m. (day 1), then one of 3 dose levels of MG1-MAGEA3 i.v. (day 15 and 18) + pembrolizumab 200 mg i.v., starting in week 6 or on day 1 (depending on cohort; intervals not reported) B: Ad-MAGEA3 fixed dose i.m. followed by pembrolizumab i.v. (day 1), then MG1-MAGEA3 i.v. (day 15) and i.t. on day 22, 29, and 36; MG1-MAGEA3 booster injections i.t. Q3W beginning at day 43 (=week 6)	AEs, maximum tolerated and feasible dose of Ad/MG-MAGEA3	ORR, DCR, PFS, DOR	Turnstone Biologics, Corp.
KEYNOTE-629 NCT03284424 (EudraCT: 2017-000594-37)	Multicenter, two-armed, open-label, phase II	Oct 2017	Jun 2022	150	Pembrolizumab 200 mg i.v. Q3W up to 2 years A: recurrent or mSCC B: laSCC	ORR	DOR, DCR, PFS, OS, AEs, discontinuations due to AEs	Merck Sharp & Dohme Corp.
KEYNOTE-630 NCT03833167 (EudraCT: 2018-001974-76)	Randomized, multicenter, blinded, controlled, phase III	Apr 2019	Sep 2027	570	Adjuvant setting A: Pembrolizumab 400 mg i.v. Q6W, up to 9 cycles; if 9 cycles completed: Up to 18 additional cycles in open-label design B: Placebo i.v. Q6W, up to 9 cycles; if disease recurrence: Up to 18 cycles of pembrolizumab in open-label design	RFS	OS, QoL, AEs, discontinuations due to AEs	Merck Sharp & Dohme Corp.
NCT02964559	Single-center, open-label, phase II	Jan 2017	Feb 2022	11	Pembrolizumab i.v. Q3W until disease progression or unacceptable toxicity	RR	OS, PFS	Emory University Merck Sharp & Dohme Corp.
CARSKIN trial NCT02883556 (EudraCT: 2016-002076-28)	Single-center, open-label, phase II	Mar 2017	Oct 2021	57	Pembrolizumab 200 mg i.v. Q3W until disease progression or unacceptable toxicity, or up to 24 months	RR	AEs, RR in PD-L1 positive patients, DCR, OS, PFS, DOR, duration of control, time to progression	Assistance Publique - Hôpitaux de Paris
NCT02721732	Single-center, open-label, phase II	Aug 2016	Aug 2020	225	Pembrolizumab i.v. Q3W until disease progression or unacceptable toxicity, or up to 24 months; responding patients may continue up to 12 additional months	Non-progression rate, AEs	ORR, clinical benefit (CR, PR or SD), DOR, PFS, OS, ECOG performance status, temperature, pulse, body weight, respiratory rate, blood pressure	M.D. Anderson Cancer Center National Cancer Institute (NCI)
NCT04234113	Multicenter, open-label, phase I	Jun 2019	Mar 2022	96	A: SO-C101 (IL-15 agonist) B: SO-C101 + Pembrolizumab i.v. unclear dosing and intervals	Dose-limiting toxicity, AEs, laboratory test abnormalities, ECOG performance status	ORR, best overall response, DOR, clinical benefit rate, PFS, anti-drug antibodies to SO-C101	Sotio a.s.

*: estimated; Abbreviations: Ad-MAGEA3: Adenovirus vaccine expressing Melanoma-associated antigen 3, AEs: adverse events, allo-HCT: allogeneic hematopoietic stem cell transplant, BID: twice daily, CR: complete response, cSCC: cutaneous squamous cell carcinoma, DCR: disease control rate, DOR: Duration of response, GVHD: Graft-versus-Host-disease, Gy: Gray, i.m.: intramuscular, i.t.: intratumoral, i.v.: intravenous, laSCC: locally advanced cSCC, MG1-MAGEA3: MG1 Maraba oncolytic virus expressing Melanoma-associated antigen 3, mSCC: metastatic cSCC, ORR: objective response rate, OS: Overall survival, PFS: Progression-free survival, p.o.: per os, PR: partial response, QoL: quality of life, QD: daily, QW: every week, Q2W: every 2 weeks, Q3W: every 3 weeks, Q4W: every 4 weeks, Q6W: every 6 weeks, RFS: recurrence free survival, RR: response rate, rhIL-7-hyFc: recombinant human interleukin-7 and hybrid Fc fusion protein, RP1: genetically modified herpes simplex virus 1; T-VEC: Talimogene laherparepvec.

## Abbreviations

α-MSH	alpha-melanocyte-stimulating hormone
ACTH	adrenocorticotropic hormone
Ad-MAGEA3	adenovirus vaccine expressing melanoma-associated antigen 3
AEs	adverse events
AK	actinic keratoses
allo-HCT	allogeneic hematopoietic stem cell transplantation
ASIP	agouti signaling protein
BCC	basal cell carcinoma
BID	twice daily
CI	confidence interval
CM	cutaneous melanoma
CR	complete response
cSCC	cutaneous squamous cell carcinoma
CTCL	cutaneous T cell lymphoma
CTL	cytotoxic T lymphocyte
DCR	disease control rate
DoR	duration of response
EAP	expanded access program
EGFR	epidermal growth factor receptor
EMA	European Medicines Agency
FDA	U.S. Food and Drug Administration
FGF	fibroblast growth factor
GVHD	graft-versus-host disease
Gy	Gray
HGF	hepatocyte growth factor
HNSCC	head and neck squamous cell carcinoma
i.m.	intramuscular
i.t.	intratumoral
i.v.	intravenous
ICB	immune checkpoint blockade
laSCC	locally advanced cSCC
MC1R	melanocortin 1 receptor
MG1-MAGEA3	MG1 Maraba oncolytic virus expressing melanoma-associated antigen 3
mSCC	metastatic cSCC
NER	nucleotide excision repair
NMSC	non-melanoma skin cancer
NSCLC	non-small cell lung cancer
ORR	objective response rate
OS	overall survival
OTR	organ transplant recipient
PD	progressive disease
PFS	progression-free survival
PR	partial response
POMC	proopiomelanocortin
PYs	person-years
Q2W	every 2 weeks
Q3W	every 3 weeks
Q4W	every 4 weeks
Q6W	every 6 weeks
QD	daily
QoL	quality of life
QW	every week
RCT	randomized-controlled trial
RFS	recurrence free survival
rhIL-7-hyFc	recombinant human interleukin-7 and hybrid Fc fusion protein
RP1	genetically modified herpes simplex virus 1
RR	response rate
SCF	stem cell factor
SD	stable disease
T-VEC	talimogene laherparepvec
XP	xeroderma pigmentosum
